# Evaluation of Antimycobacterial Activity of Higenamine Using *Galleria mellonella* as an In Vivo Infection Model

**DOI:** 10.1007/s13659-018-0152-3

**Published:** 2018-01-22

**Authors:** Paul Erasto, Justin Omolo, Richard Sunguruma, Joan J. Munissi, Victor Wiketye, Charles de Konig, Atallah F. Ahmed

**Affiliations:** 1 National Institute for Medical Research, P.O. Box 9653, Dar Es Salaam, Tanzania; 2 Department of Chemistry, University of Dar es Salaam, Dar Es Salaam, Tanzania; 3 School of Chemistry, Witwatersrand University, Johannesburg, Republic of South Africa; 4 Department of Pharmacognosy, College of Pharmacy, King Saud University, P.O. Box 2457, Riyadh, 11451 Saudi Arabia

**Keywords:** *Aristolochia brasiliensis*, *Galleria mellonella*, Higenamine, Isoniazid, *Mycobacterium indicus pranii*, Antimycobacterial activity

## Abstract

The Phytochemical investigation on MeOH extract on the bark of *Aristolochia brasiliensis* Mart. & Zucc (Aristolochiaceae) led to the isolation of major compound (**1**) as light brown grainy crystals. The compound was identified as 1-(4-hydroxybenzyl)-1,2,3,4-tetrahydroisoquinoline-6,7-diol (higenamine) on the basis of spectroscopic analysis, including 1D and 2D NMR spectroscopy. The compound was evaluated for its antimycobacterial activity against *Mycobacterium indicus pranii* (MIP), using *Galleria mellonella* larva as an in vivo infection model. The survival of MIP infected larvae after a single dose treatment of 100 mg/kg body weight of higenamine was 80% after 24 h. Quantitatively the compound exhibited a dose dependent activity, as evidenced by the reduction of colony density from 10^5^ to 10^3^ CFU for test concentrations of 50, 100, 150 and 200 mg/kg body weight respectively. The IC_50_ value for higenamine was 161.6 mg/kg body weight as calculated from a calibration curve. Further analysis showed that, a complete inhibition of MIP in the *G. mellonella* could be achieved at 334 mg/kg body weight. Despite the fact that MIP has been found to be highly resistant against isoniazid (INH) in an in vitro assay model, in this study the microbe was highly susceptible to this standard anti-TB drug. The isolation of higenamine from the genus *Aristolochia* and the method used to evaluate its in vivo antimycobacterial activity in *G. mellonella* are herein reported for the first time.

## Introduction

The mycobacterial infections continue to be among the leading causes of morbidity and mortality in the developing countries. The situation is further exacerbated by the increasing cases of drug resistant mycobacteria strains which complicates the treatment programmes. This has compelled for a global renewal of strategies to search for new or modified antimycobacterial agents effective against drug resistant strains [1, 2].

Medicinal plants present a reservoir of antimycobacterial compounds with varying structures worthy for further exploration. Plants species such as those in the genus *Aristolochia* are ethnomedically used in the treatment of various bacterial infections and other ailments in Asia, Latin America. The genus *Aristolochia* has over 500 species [3], thus offers various medicinal applications in different parts of the world [4–6]. Although *Aristolochia* species contains aristolochic acids which have been reported to be nephrotoxic [7–9], they however contain other secondary metabolites which have various pharmacological importance. For example, protoberberins, isoquinolines, benzylisoquinolines, flavonoids, lignans and coumarins among many other classes of compounds have various pharmacological values [10–14].

In Tanzania, *Aristolochia brasiliensis* Mart. & Zucc (Aristolochiaceae) is used in the treatment of chronic and persistent coughs around Lake Victoria basin in Tanzania. It is further used as a herbal supplement for improving immune system in immunocompromised patients particularly those infected with HIV/AIDS and tuberculosis (unpublished ethnomedical information). In our effort to search for antimycobacterial alkaloids, a hydroxybenzylisoquinoline also known as Higenamine was isolated from the leaves of *A. brasiliensis*. Its chemical structure was deduced through NMR, MS and UV spectroscopic analyses. Despite of its existence in other plant genera, the isolation of Higenamine from this plant species is herein reported in this genus and species for the first time. Higenamine was evaluated for in vivo antimycobacterial efficacy using *Galleria mellonella,* a sixth instar larva of a great wax moth as an in vivo infection model, while *Mycobacterium indicus pranii* (MIP) was used as a test microorganism in the assay. The choice of MIP was based on the fact that it is highly resistant against isoniazid in an in vitro assay model [15–17], thus providing a potential surrogate resistant mycobacteria bacilli for this study.

## Results and Discussion

### Structural Elucidation of Compound 1

**Fig. 1:**
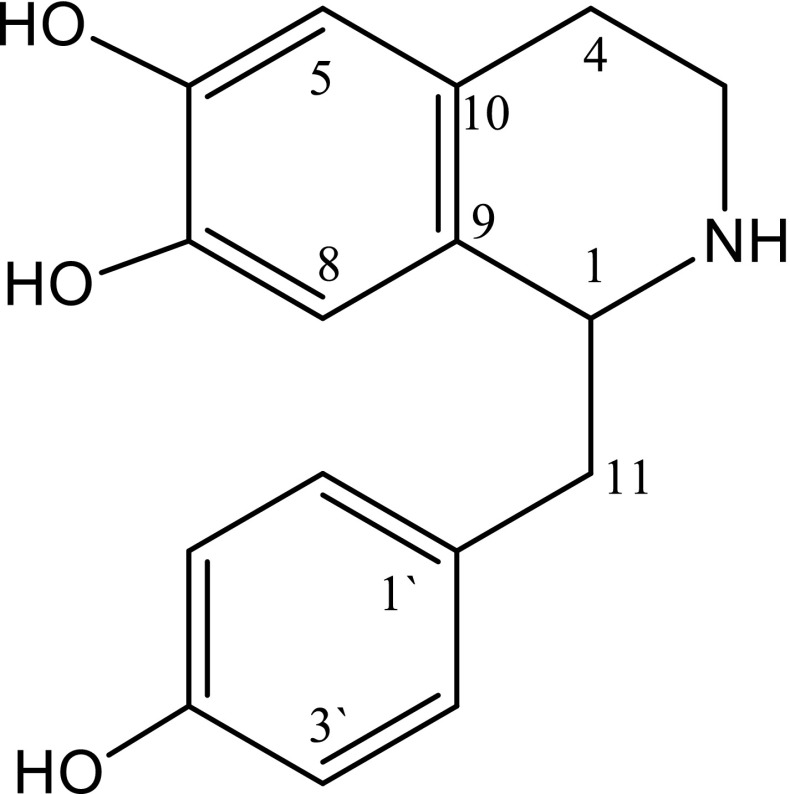
The chemical structure of compound **1** isolated from the leaves of *Aristolochia brasiliensis*

The EI-MS and NMR spectral data suggested a molecular formula C_16_H_17_NO_3_ for compound **1** and nine degrees of unsaturation in the molecule. The ^13^C NMR spectrum measured in CD_3_OD, with the assistance of DEPT and HSQC experiments, revealed the presence of 12 *sp*^2^ carbon signals of two phenyl moieties (*δ*_C_ 158.3–114.3 ppm), three *sp*^3^ methylenes (*δ*_C_ 41.0–25.9 ppm), and a *sp*^3^ methine group (*δ*_C_ 58.0). Therefore, the remaining degree of unsaturation are ascribable for a heterocyclic ring containing the nitrogen atom. Moreover, the ^1^H NMR spectrum of compound **1** showed the presence of two 2H doublets at *δ*_H_ 7.12 and 6.80 (each d, *J* = 8.0 Hz) and two 1H singlets at *δ*_H_ 6.62 and 6.63 of 1,4-di- and 1,2,4,5-tetrasubstituted phenyls. Furthermore, on the connectivities of the *sp*^3^ carbons with the nitrogen atom and the two phenyl moieties were established by the analysis of correlations found in the ^1^H-^1^H COSY and HMBC spectra and hence the final structure of the molecule as shown in Fig. 1. The compound was thus identified as higenamine.

Higenamine was first isolated in 1978 from the roots of *Aconitum japonicum* THUMB [18]. The extracts of this medicinal plant was used as an important heart stimulant, diuretic agent and anodyne among many other uses in Chinese medicine [18]. The compound has further been reported from several other plant species namely *Nandina domestica, Tinospora crispa, Lateralis Preparata, Nelumbo nucifera* [19–21]. From the genus *Aristolochia*, as well as the plant species *A. brasiliensis*, this is the first report, thus expanding our knowledge of Higenamine existence in nature from various Genera.

### In Vivo Antimycobacterial Activity of Higenamine

The choice of *G. mellonella* as an insect model system in this study has a significant advantage over other alternatives because the larva can be incubated at human core temperature of 37 °C and has immunological responses which can easily be observed and recorded [22, 23]. Despite being reported previously in various literature, the use of *G. mellonella* as a host for mycobacterial infections has never been documented elsewhere, thus it is herein reported for the first time. Furthermore the in vivo assay design and analyses of results used in this study are also new and herein reported for the first time.

### Survival and Behavioral Assay of *Galleria mellonella*

**Fig. 2:**
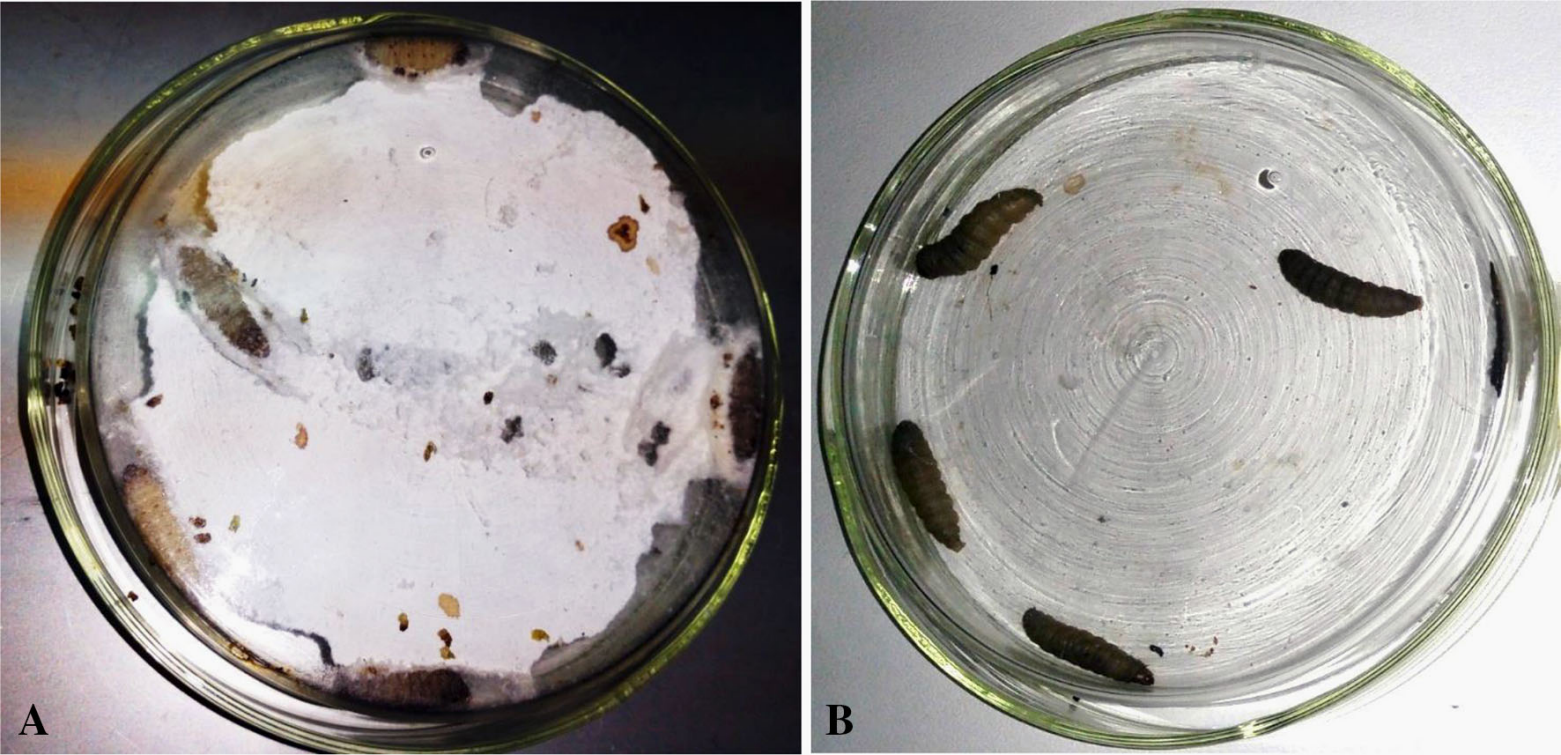
**a** *Galleria mellonella* larvae injected with sterile 7H9 TB broth, showing typical behavior of a health larva, by chewing a filter paper and producing silk for their territories (negative control). **b** The positive control cohort of *Galleria Mellonella* which was injected with *Mycobacterium indicus pranii*, showing a clear change of haemocyte color due to infection, and unable to produce silk

The survival assay was evaluated by injecting five MIP infected larvae with 100 mg/kg body weight of higenamine. These were compared with two control groups which comprised of five infected larvae but not treated, and five larvae received normal saline and blank 7H9 TB broth. Results show that, 80% of the MIP infected larvae which received 100 mg/kg of higenamine survived after 24 h. This implied that only one larva died, equivalent to 20%. On the other hand, the negative control group had 100% mortality within 24 h due to heavy MIP infection, while all larvae in the control group injected with 20 µL of sterile 7H9 broth followed by 20 µL of blank normal saline, survived after 24 h (Fig. 2a, b). Normally, healthy *G. mellonella* larva produces silk as a way of creating protective territory for themselves from external threats. A direct observation on the behavior of larva infected with MIP then treated and/or not treated with a bioactive compound provide important clue about the efficacy of the test secondary metabolite. For example, in this assay the larvae in the control (normal control) group lost the ability to produce silk indicating that, the MIP infection in their bodies was heavy compared to the higenamine treated and negative control cohorts (Fig. 2a, b). This is a direct observation on the efficacy of the compound against MIP tested in an in vivo assay model.

### Comparison of MIP Colony Density Between Higenamine Treated and Untreated *Galleria mellonella* Larva

**Fig. 3:**
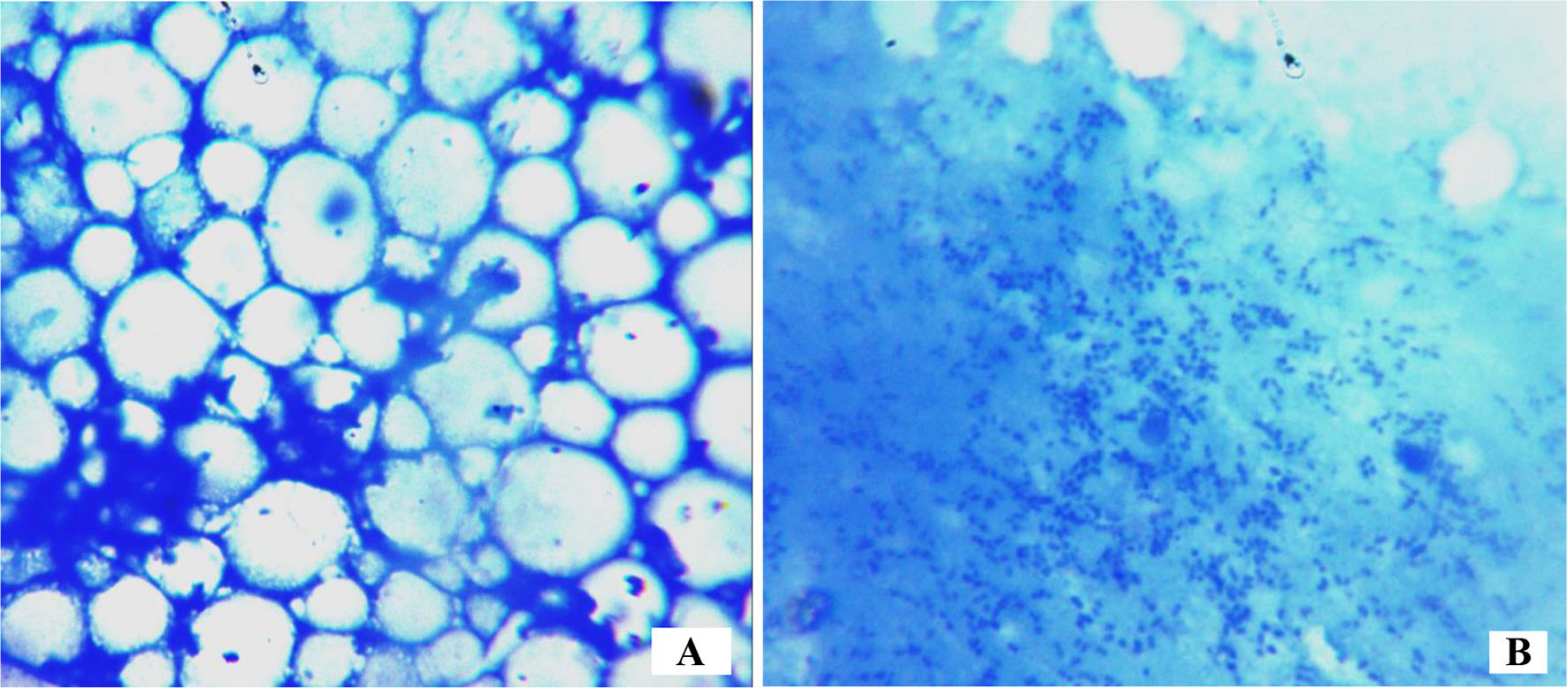
Non-infected healthy haemocyte cells in *G. mellonella* larva (normal control,** a**) and destructed cells with a heavy proliferation of MIP bacilli in the infected *G. mellonella* larva (negative control,** b**)

The clear differences of cell distribution patterns between the treated, positive and negative control groups was observed through colony densities taken from different fields. The microscopic observation of the haemocyte cells of larvae not infected with MIP (normal control) showed healthy and well distributed cells across the slide, while that of the infected larvae and not treated with any drug (negative control) showed a heavy colony density and complete destruction of the cells and cellular pattern (Fig. 3a, b).

**Fig. 4:**
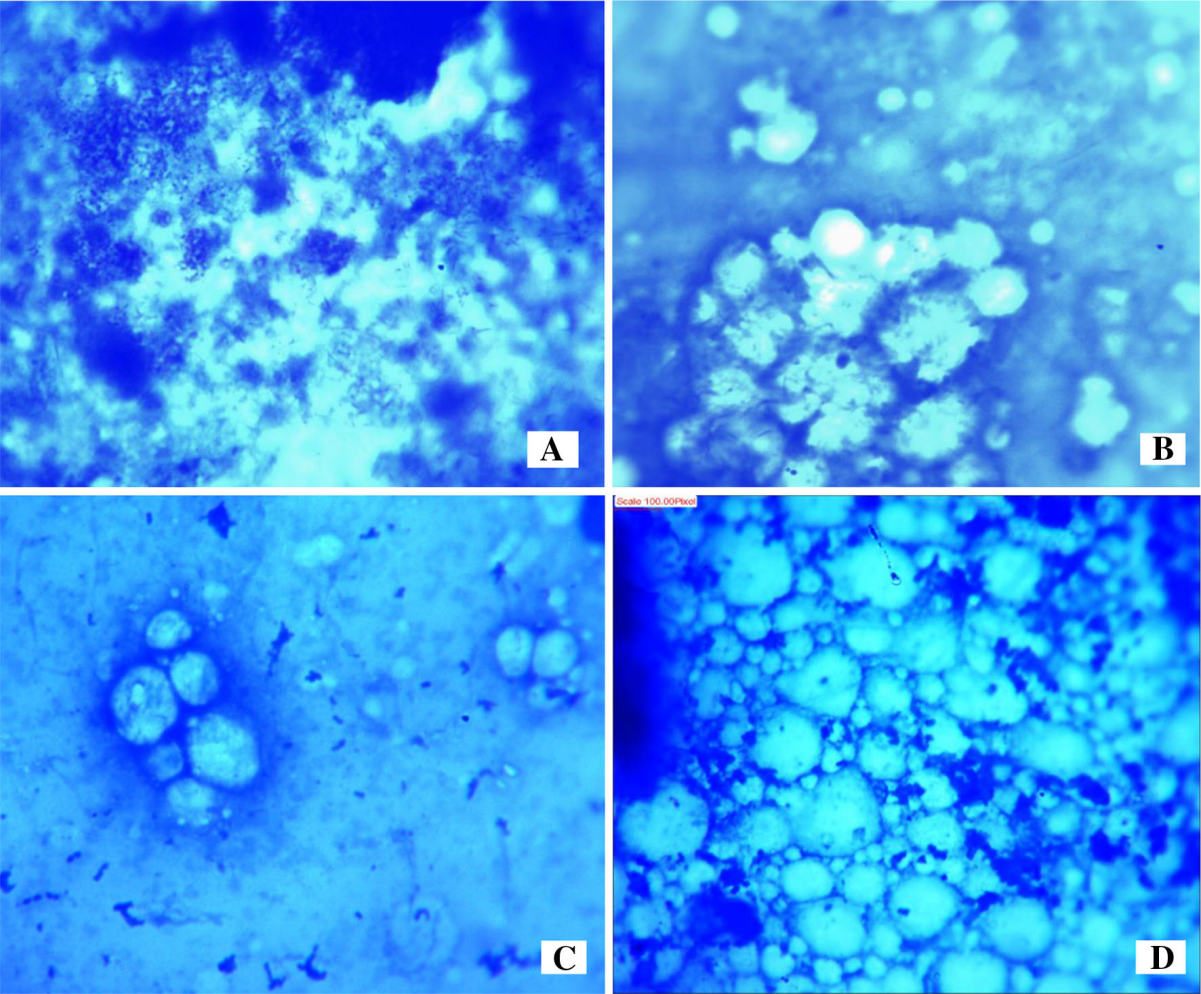
Microscopic analysis of MIP infected haemocyte cells in *G. mellonella* larva following the treatment with higenamine at 50 mg/kg (**a**), 100 mg/kg (**b**), 150 mg/kg (**c**) and 200 mg/kg (**d**) per body weight, respectively

In the cohorts treated with higenamine, the distribution of healthy cells and their pattern varied with varying concentrations. It was observed that, as the concentration of the compound increased, more cells were protected (Fig. 4a, b, c, d). Further observations showed some healthy cells being surrounded by the MIP bacilli. This was evident that, higenamine has the ability to deter mycobacteria from entering the cells (Fig. 4c, d). It further imply that, the ability of the compound to penetrate the cells of the larva is an important factor in the elicitation of its efficacy. Generally, the colony densities ranged from slight to moderate (10^3^–10^5^ CFU), and it was dose dependent. These observations show that, the preliminary evaluation of in vivo antimycobacterial activity of compounds can be determined qualitatively to provide important clue on their efficacy prior to conducting quantitative bioassay.

### Quantitative Antimycobacterial Activity of Higenamine in *Galleria mellonella*

**Fig. 5:**
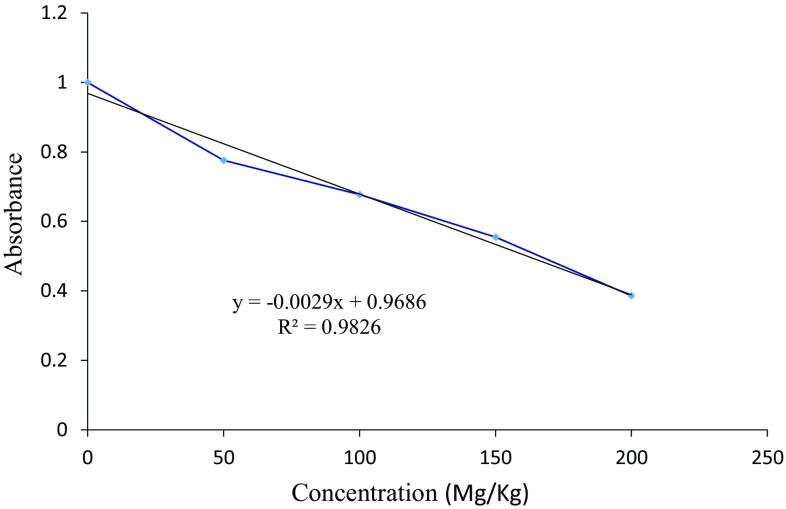
Quantitative in vivo antimycobacterial evaluation of higenamine against MIP infected *G. mellonella* larvae as an infection model

**Fig. 6:**
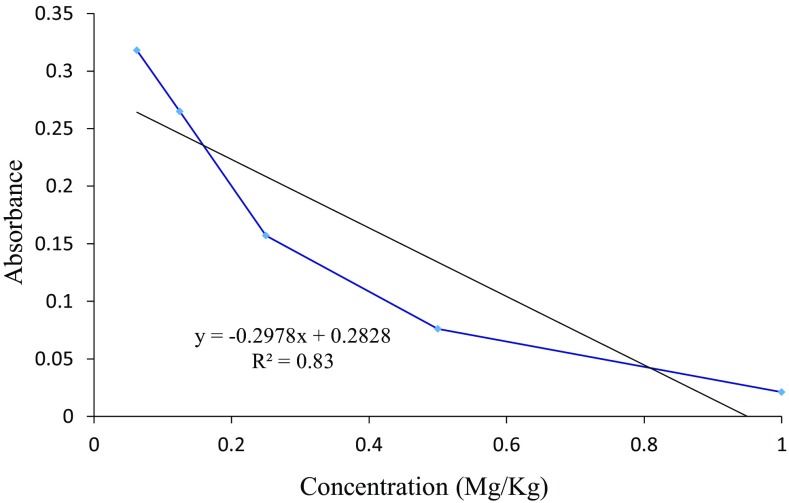
In vivo antimycbacterial activity of Isoniazid, a standard anti-TB drug screened against MIP infected *G. mellonella* larva as an infection model

The quantitative antimycobacterial efficacy of higenamine was determined by plotting the test concentrations against their respective absorbance. This was after sub-culturing the MIP infected haemocyte cells in the 7H9 TB broth for 24 h. The guiding principle in this assay is that, if the compound is active, there should be none or few active MIP cells in the sub-cultured sample, therefore the metabolism of INT is low implying low absorbance (lower concentration of produced formazan). Whereas, if the compound has weak or no activity, the proliferation of MIP in the sub-cultured sample will be high, therefore the metabolism of INT will also be higher giving higher amount of formazan, implying higher absorbance. Therefore, the higher the absorbance the weaker bioactive the compound or drug is, and vice versa. A typical plot of the highly active compound against a test microbe, will have a negative gradient indicating growth inhibition and thus weak INT metabolism by the surviving microbe cells. Based on this background, higenamine exhibited a dose dependent in vivo antimycobacterial activity against MIP. As shown in Fig. 5, the absorbance decreased with increasing the concentration of the test compound. A similar trend was observed for the standard drug, isoniazid (INH) which exhibited a dose dependent efficacy, though at a much lower concentration levels (Fig. 6). An extrapolation from Fig. 5 revealed the IC_50_ value of higenamine as 161.6 mg/kg body weight calculated from the equation Y = − 0.0029x + 0.9686. Whereas a complete inhibition of MIP growth in the *G. mellonella* larva body could be achieved at a single dose treatment of 334 mg/kg body weight.

Higenamine, is a benzylisoquinoline alkaloid, a group of compounds which have been studied extensively for antimycobacterial activity. Various reports indicates that, quinolines and isoquinolines have higher antimycobacterial activities thus providing important clue for further chemical structure modifications leading to drug discovery [17, 24]. This study has revealed further that, testing natural products in an in vivo model provides more options for analysis and inferences. Additionally, the assay has shown that, MIP is highly susceptible to isoniazid (INH) when tested in an in vivo assay model, however, when tested in an in vitro assay, isoniazid lacks efficacy against this *Mycobacterium* species even at higher concentrations [1, 15–17]. This observation has provided further opportunity to investigate the reasons why in the in vivo assay model INH was highly efficacious while when tested in the in vitro assays it lacks efficacy against MIP even at higher concentrations.

## Materials and Methods

### General Analyses

The 1-D and 2-D NMR data of Higenamine was obtained using Bruker Avance Ultrashield 400 Plus NMR machine operating at a spectrometer frequency of 400 MHz for ^1^H-NMR and 75 MHz for ^13^C-NMR. EI-MS spectra were recorded on a Finnigan MAT SSQ 7000 Single Quadrupole Instrument. UV spectroscopic analysis was done using an S2000 UV/Vis Diode Array Spectrophotometer, Biochrom machine while melting points were measured on a Stuart Scientific (SMP1) melting point apparatus. The varying colony density of *M. indicus pranii* in the *G. mellonella* haemocytes was qualitatively evaluated under microscope at ×100 oil immersion. The quantitative evaluation of in vivo antimycobacterial efficacy of Higenamine was done spectrophotometrically using an S2000 UV/Vis Diode Array Spectrophotometer, Biochrom machine.

### Chemicals

All solvents were purchased from Carlo Erba (France), Middlebrook 7H9 TB broth base was obtained from HIMEDIA (India), Glycerol (AR) obtained from Lab Equip Ltd (Tanzania), iodonitrotetrazolium (INT) chloride, Isoniazid (R&D) were purchased from Sigma (UK). Silica gel Kiesegel 60 PF_254_ and pre-coated Aluminium backed silica gel 60 F-_254_ (0.2 mm thickness) TLC plates used during isolation of higenamine were obtained from Merck South Africa Pty.

### Collection, Preparation and Extraction of Plant Materials

The leaves of *A. brasiliensis* were collected from Geita Town, Geita Region, Tanzania. Identification was done on site by the plant taxonomist. The leaves of *A. brasiliensis* were air dried under shade for 10 days and thereafter pulverized into powder using an electric miller. 500 g of the powdered leaves of *A. brasiliensis* were soaked in methanol three times, each round for 24 h followed by concentration in *vacuo* using a rotary evaporator. This afford 60.4 g of crude extracts. The extract was kept in the refrigerator (4 °C) ready for chromatographic isolation of compounds.

### Isolation of Higenamine from the Leaves of *A. brasiliensis*

The methanol extract (25.4 g) from the stem bark of *A. brasiliensis* was adsorbed in silica gel and loaded on a silica gel column eluting with dichloromethane:Methanol with increasing polarity from 9:1 to 7:3 respectively. A total of 35 fractions each with 150 ml of eluates were collected. After TLC analysis, fractions 1–8, 9–18, 19–30 and 31–35 were combined. Further TLC analyses on fractions 1–8 and 9–18 revealed no alkaloids after spraying with Dragendorf reagent, therefore were discarded. The TLC analysis of fraction 19–30 revealed the presence of alkaloids and therefore was subjected on further column chromatographic separation. The fraction was adsorbed in silica gel and loaded on the silica gel column eluting with CH_2_Cl_2_:MeOH (8.5:1.5) to give 30 sub-fractions each with 50 ml of eluates. After TLC analysis, sub-fractions 1–14 were discarded, while sub-fractions 15–22, and 23–30 were combined and left under the hood overnight. From sub-fractions 15–22 and 23–30 creamy colored grain like crystals formed around the walls of the flasks. The crystals were collected and washed using 100% dichloromethane and left to dry overnight under the fume hood to yield 256 mg of cream grainy crystals ready for spectroscopic analyses and structural identification.

#### Spectroscopic Data of Higenamine

Higenamine (**1**): Light brown grainy crystals (256 mg); m.p.: 258–260 °C, EI-MS: *m/z* 271. (10%, M^+^) calculated for C_16_H_17_NO_3_, 164 (100%), 149 (60%). UV (MeOH) λ_max_ (logε) nm 227 (4.23), 285 (3.72). ^1^H NMR (500 MHz; CD_3_OD): *δ*7.12 (2H, d, *J* = 8.0 Hz, H-2′ & H-6′), 6.80 (2H, d, *J* = 8.0 Hz, H-3′ & H-5′), 6.61 (1H, s, H-8), 6.62 (1H, s, H-5), 4.56 (1H, m, H-1), 3.45 (1H, m, H-3b), 3.35 (1H, m, H-11b), 3.24 (1H, m, H-3a), 2.98 (2H, m, H-4b & H-11a), 2.90 (1H, m, H-4a). ^13^C NMR (125 MHz; CD_3_OD): *δ*158.3 (C, C-4′), 146.9 (C, C-6), 145.8 (C, C-7), 131.7 (2 CH, C-2′& C-6′), 127.1 (C, C-1′), 123.9 (2C, C-9 and C-10), 117.0 (2 CH, C-3′& C-5′), 116.3 (CH, C-8), 114.3 (CH, C-5), 58.0 (CH, C-1), 41.0 (CH_2_, C-3), 40.6 (CH_2_, C-11), 25.9 (CH_2_, C-4).

### Antimycobacterial Screening

#### Test Organism

The mycobacteria strain, namely *Mycobacterium indicus pranii* (MIP) DSM 45239 was supplied by the Germany Resource Centre for Biological Materials, Braunschweig, Germany. This is an acid-fast growing and isoniazid resistant mycobacteria strains. It was used as a surrogate drug resistant mycobacteria bacilli for determination of a potential anti-TB efficacy of Higenamine in an in vivo assay model. MIP was sub-cultured in glycerol supplemented 7H9 TB broth as described by Erasto et al. [1, 17]. The *Galleria mellonella* sixth instar larvae of great wax moth were obtained from bee keepers in Msata Village, Bagamoyo districts, Tanzania. In order to avoid interaction with outside environment after collection, the larvae were maintained in the bee combs prior to their use in the assay.

#### Survival and Behavioral Assay for the *Galleria mellonella* Treated with Higenamine

The larva were inoculated by injecting 20 µL of broth containing MIP and left for two hours before being treated with 100 mg/kg of Higenamine solution. The injections were done as described by Wand et al. [25]. The treatment was replicated thrice with each petri-dish containing 5 larvae. The number of dead and alive *G. mellonella* larva was counted 24 h after treatment. Thereafter the percentage of survival and dead larva was determined. Furthermore, the behavioral change between treated and untreated cohorts was observed and recorded.

#### Qualitative and Quantitative Determination of Efficacy of Higenamine Against

##### *Mycobacterium indicus pranii* in *Galleria mellonella*

In order to determine the in vivo efficacy of Higenamine quantitatively, serial dilutions from 200 mg/kg to 50 mg/kg body weight were prepared giving four test concentrations namely; 50, 100, 150 and 200 mg/kg. For each test concentration two replicates of three larvae each weighing an average weight of 280 ± 10 mg were placed in a sterile petri dish ready for inoculation and treatment. A full grown culture of MIP was centrifuged to suspend the colonies evenly in the broth, followed by injecting 20 µL of innocula into *G. mellonella* body. While the control group larvae were injected with 20 µL of normal saline. The larvae were incubated at 37 °C in the incubator for 2 h to allow the mycobacteria infection to pick-up. This was observed through immunological response of the larva where it changed its color from cream to dark. Thereafter, the larva were treated separately with 50, 100, 150 and 200 mg/kg of Higenamine respectively, while the negative and normal control groups were injected with another 20 µL of normal saline and incubated at 37 °C for 24 h prior to further analyses. After 24 h, the count of live and dead larva was conducted to obtain the mortality rate, and the behavioral observation of the larva in all the treatments was also recorded.

For further qualitative and quantitative analysis, one live larva from each treatment was dissected to obtain a portion of the haemocyte and fluids for smearing on a slide for microscopic analysis and imaging to determine the colony density. The remaining whole haemocyte was crushed and transferred into 2 mL of sterile broth and centrifuged (×10000) to suspend MIP colonies. Thereafter, 100 µL was transferred into pre-prepared sterile vials containing 5 mL of 7H9 TB broth. This followed by incubation of the vials for 24 h. Thereafter three replicates containing 1 mL of the each culture were drawn followed by addition of 100 µL of 0.2 mg/mL of Iodonitrotetrazolium salt (INT). The mixtures were incubated at 37 °C for 2 h. One negative and normal control replicates (i.e. larvae treated with normal saline and blank sterile broth, and those infected with MIP but not treated with the test drug) were used as a negative and normal controls respectively.

The quantitative in vivo antimycobacterial efficacy of Higenamine against MIP was determined by scanning each treated replica on a UV/Vis diode array spectrophotometer, Biochrom set at a UV wavelength of 380 nm. The spectrophotometer determined the amount of a UV absorbing formazan formed as a result of INT metabolism by the active MIP cells. The amount of MIP cell available in the broth is proportional to the absorbance, hence the lower the absorbance the fewer the active MIP cells and vice versa. This gave activity curves of absorbance versus concentration (mg/kg). The quantity of Higenamine required to completely inhibit the growth of MIP in the *G. mellonella* was determined by extrapolation. Following the same procedure, isoniazid a standard anti-TB drug was tested at the concentration of 0.2, 0.4, 0.6, 0.8 and 1.0 mg/kg body weight. The observations were recorded as described in the tests above.
